# Deduction of full factorial design of HPLC technique for the simultaneous analysis of meloxicam and esomeprazole in their laboratory prepared tablets

**DOI:** 10.1038/s41598-025-95706-3

**Published:** 2025-04-15

**Authors:** Hesham Sameh Ramadan, Fathalla Belal, Aya Roshdy, Mohamed M. Salim

**Affiliations:** 1 Department of Pharmaceutical Analytical Chemistry, Faculty of Pharmacy, Suez Canal University, Ismailia, Egypt; 2 Department of Pharmaceutical Chemistry, Faculty of Pharmacy, Horus University- Egypt, New Damietta, Egypt; 3 Department of Pharmaceutical Analytical Chemistry, Faculty of Pharmacy, Mansoura University, Mansoura, 35516 Egypt

**Keywords:** Meloxicam, Esomeprazole, HPLC, Factorial design, Pharmaceutical tablets, Analytical chemistry, Green chemistry

## Abstract

A reversed phase HPLC method with UV detection was designed for the first time for the simultaneous estimation of meloxicam and esomeprazole in their combined tablet dosage forms. Full factorial design was used for rapid optimization of the proposed method. Chromatographic separation was reached using isocratic elution on C18 column. The mobile phase was a mixture of methanol: acetonitrile: 0.05 M potassium dihydrogen phosphate buffer at pH 5, adjusted using phosphoric acid and/or NaOH when needed. The flow rate was 1 mL/min and the injection volume was 20 µL. The detection wavelength was 230 nm. The working ranges of the method were 5.0-100.0 and 10.0–100.0 µg/mL, LOD values were 0.8 and 1.8 µg/mL and LOQ values were 2.6 and 5.5 µg/mL for meloxicam and esomeprazole, respectively. The proposed method was successfully applied to their combined tablet dosage forms with acceptable % recoveries (100.4 − 100.7%) obtained. Four methods were used to evaluate the greenness of the proposed method, suggesting the acceptability of the greenness of the proposed method.

**Supplementary Information:**

The online version contains supplementary material available at 10.1038/s41598-025-95706-3.

## Introduction

**Fig. 1:**
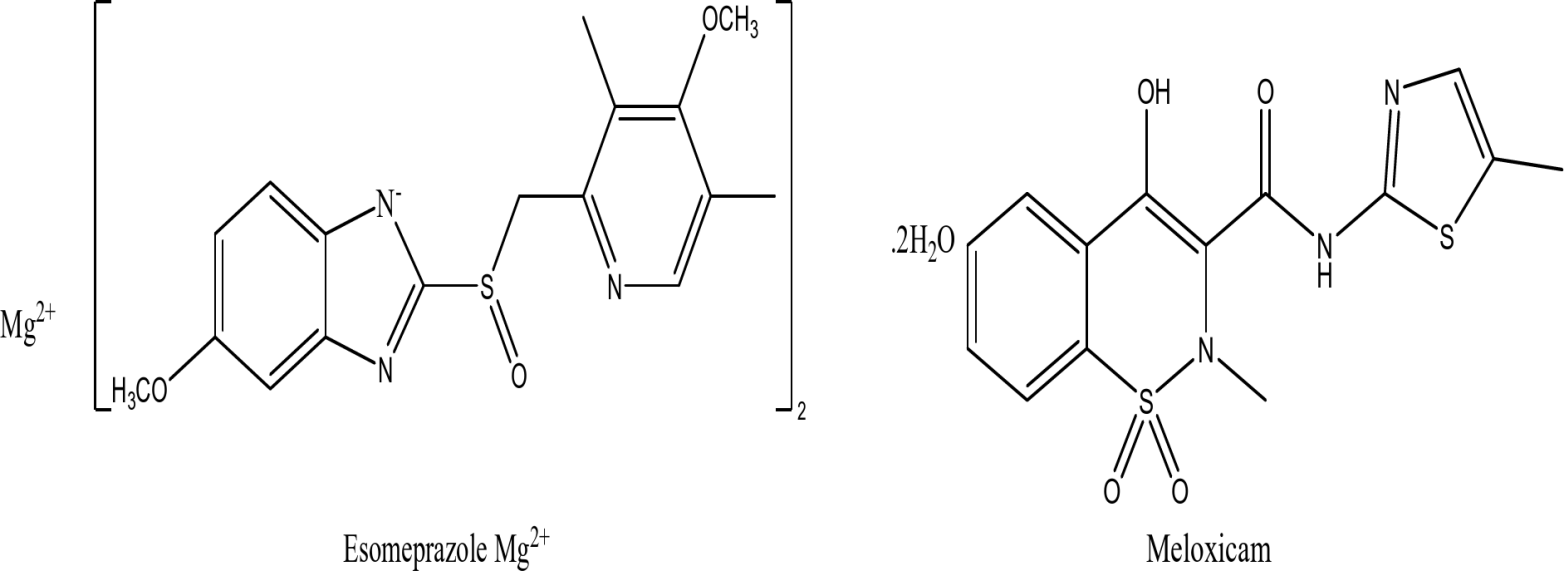
chemical structure of the studied compounds.

Meloxicam (MEL) (Fig. 1) (pKa 1.1, 4.2, soluble in dimethylformamide and insoluble in water) is a selective COX-2 inhibitor, with anti‑inflammatory, analgesic and antipyretic properties, having the advantageous property of being gastric mucosal and renal protective drug, is thus mainly used for osteoarthritis, rheumatoid arthritis, ankylosing spondylitis, in addition to various pain conditions of skeletomuscular origin^1^. Reports conclude that no or minimal risk of myocardial or renal outcomes are associated with MEL^2^. MEL is official in both the British Pharmacopoeia^3^ and the United States Pharmacopoeia^4^. Several methods of analysis for MEL alone or with other drugs have been reported in the literature including HPLC^5–7^, TLC^5,8^, spectrophotometry^9^, spectrofluorimetry^10–14^, electrochemical methods^15–19^, LC-MS^20–22^ and Capillary electrophoresis^23^.

Esomeprazole (EPL) (Fig. 1) (pKa 2.53, 4.44, 9.71, soluble in methanol and slightly soluble in water) is a proton pump inhibitor (PPI) and the S-isomer of omeprazole, the first to be developed as a single optical isomer. It reduces gastric acid secretion by binding to hydrogen–potassium adenosine triphosphatase pump (H+,K+-ATPase) in gastric parietal cells. The drug efficacy was validated in different gastrointestinal diseases including gastro-esophageal reflux disease (GERD) and Zollinger-Ellison’s syndrome in addition to being used in combinations for treating *Helicobacter pylori* infections and counteracting gastrointestinal adverse effects including dyspepsia, abdominal pain and ulcer lesions associated with NSAIDs^24^. It is official in the British Pharmacopoeia^3^ and the United States Pharmacopoeia^4^. Regarding its analysis whether alone or in combinations, numerous methods were reported including HPLC^25–27^, TLC^27–29^, spectrophotometry^30–33^, spectrofluorimetry^34^, capillary electrophoresis^35,36^, electrochemical methods^37–41^, HRMS^42^ and supercritical fluid chromatography^43^.

MEL/EPL combination is approved as coated tablets and capsules as an anti-inflammatory for patients having high risk of developing peptic ulcer, with other alternative treatment options deemed inadequate^44^. The combination is authorized under the brand name (OCAM PROTECT) in Ecuador and Colombia^44,45^.

Experimental design is largely favored nowadays upon devising an HPLC method for the analysis due to the presence of several factors controlling the process of separation, rendering the process of optimization laborious and tedious if method optimization is performed using the traditional univariate process, i.e., varying the values of one parameter while keeping the others constant. On the other hand, using experimental design allows for a definite number of experiments and easy optimization utilizing available software programs, effectively saving time, cost and effort^46^. Several papers were published utilizing HPLC with experimental design^47–50^.

The current work describes an HPLC method for the first time utilizing full factorial design for the simultaneous estimation of MEL and EPL in their combined tablets. To the best of our knowledge, no analytical methods were yet reported for the determination of MEL/EPL combination.

## Experimental

### Apparatus

-AZURA^®^ Analytical HPLC system (Knauer Corp., Germany) having an AZURA^®^ Pump P 6.1 L HGP, DGU-20 A5 degasser, CBM-20 A interface and AZURA^®^ UVD 2.1 L detector. Rheodyne injector valve and 0.45 μm membrane filters (Millipore, Cork, Ireland).
-The chromatographic data obtained were manipulated using Perkin Elmer TM Series Software.
-pH-27B Benchtop pH Meter (Acculab USA) was used for pH measurement.
-Minitab^®^ statistical software was utilized for the factorial design and statistical analysis (release 16 for windows, state college, Pennsylvania, USA).

### Materials and drugs

**-**Esomeprazole was obtained from Astra Zeneca Company (Cairo, Egypt), with labelled purity of 99.8%.
-Meloxicam was obtained from Amriya Pharmaceutical Industries (Alexandria, Egypt), with claimed purity of 99.8%.
-Mobitil^®^ tablets containing 15 mg MEL was purchased from the local market (Medical Union Pharmaceuticals, Cairo, Egypt).
-Esmopump^®^ tablets containing 20 mg EPL was purchased from the local market (Organo MS Pharma, Cairo, Egypt).

### Solvents and reagents

-Methanol & acetonitrile were of HPLC grade. Methanol was obtained from Lichrosolv (Supelco, Germany) and acetonitrile was obtained from Sigma-Aldrich (Germany).
-Potassium dihydrogen phosphate (KH_2_PO_4_) was obtained from Fischer Scientific and sodium hydroxide (NaOH), was obtained from ADWIC Co. (Cairo, Egypt).

### Standard solutions

Stock solutions of EPL and MEL were prepared separately by weighing 10.0 mg then dissolving in methanol using 100 mL-volumetric flasks to obtain solutions of concentrations 100.0 µg/mL of each drug. Further working solutions were diluted using methanol in 10 mL-volumetric flasks as needed. The stock solutions were stable up to 2 weeks upon refrigeration.

### Mobile phase

The used mobile phase is a mixture of 20 mL methanol, 20 mL acetonitrile and 60 mL 0.05 M KH_2_PO_4_ adjusted to pH 5 using 0.2 M NaOH and/or phosphoric acid when needed. Prior to use, the solution was subjected to ultrasonication and filtration using 0.45 μm membrane filters.

### Procedure

#### Chromatographic conditions

The injection volume for each sample was 20 µL, the flow rate was 1 mL/min and the detection wavelength was set at 230 nm at ambient column temperature and the column used was Phenomenex luna C18 (150 mm× 3 mm i.d., 3 μm particle size).

#### Construction of calibration graphs

Different volumes of stock solutions of both drugs were separately transferred into 10 mL volumetric flasks and diluted with methanol. Concentrations used for MEL were 5.0, 10.0, 30.0, 50.0, 70.0 and 100.0 µg/mL and concentrations used for EPL were 10.0, 30.0, 50.0, 70.0 and 100.0 µg/mL. Each sample was analyzed in triplicates and the average peak area was plotted against the concentration of each sample to construct the calibration curve and the corresponding regression equations were obtained. Synthetic mixtures of both drugs in the ratios of (5.0:6.67, 10:13.33, 30:40, 50:66.67 and 70:93.33 MEL to EPL, respectively) were prepared by transferring suitable aliquots from the stock solution and then completing to the mark with methanol in 10 mL-volumetric flasks. The mixtures were analyzed in the same manner.

#### Analysis of laboratory prepared tablets

The dosage form contains MEL and EPL in the ratio of 15 to 20, respectively and is only commercially available in each of Ecuador and Colombia^44,45^. As a result, laboratory prepared tablets were used for analysis. Ten tablets of each of Mobitil^®^ & Esmopump^®^ were ground separately in mortars then, weighed amounts equivalent to 15 mg MEL and 20 mg EPL, respectively were further ground together in another mortar, simulating the actual dosage form. The ground powder was transferred into 100 mL-volumetric flask and around 60 mL aliquot of methanol was added. The flask was subjected to sonication for 30 min, then the volume was completed to 100 mL with methanol. The solution was then filtered and appropriate volumes were withdrawn and diluted with methanol in 10 mL-volumetric flasks to reach the following ratios: (15:20, 20:26.7, 25:33.3 & 30:40) µg/mL MEL to EPL, respectively.

## Results and discussion

### Optimizing the experimental parameters

#### Column choice

Three columns were tested, namely Phenomenex luna C18 (150 mm× 3 mm i.d., 3 μm particle size), Cyano column (250 mm× 4.6 mm i.d., 5 μm particle size) and Nucleosil 100-5 Phenyl column (250 mm× 4.6 mm i.d., 5 μm particle size). The column of choice was Phenomenex luna C18 as it produced distinct peaks with acceptable resolution, with the other columns failing in peaks separation.

#### Detector wavelength

**Fig. 2:**
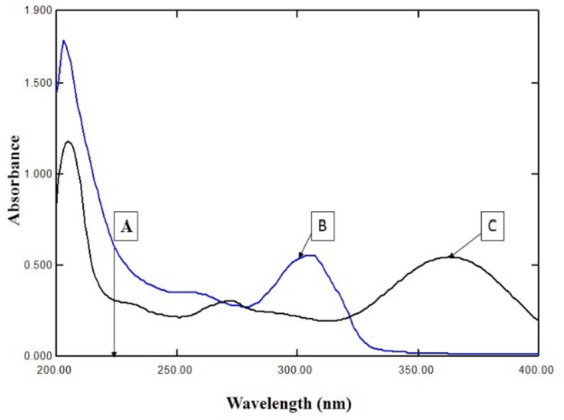
UV spectra of MEL (**C**) and EPL (**B**) (10.0 µg/mL), Point **A **is the chosen wavelength at 230 nm.

Upon literature survey and after examining the UV spectra of both drugs, it was found that the wavelength that would be suitable for both drugs is 230 nm, so 230 nm was chosen as it gave satisfactory intensity for both drugs (Fig. 2).

#### The mobile phase composition and pH

Initial trials revealed that using only methanol or acetonitrile with potassium dihydrogen phosphate KH_2_PO_4_ buffer at different pHs failed in the separation of two drugs resulting in very broad peaks with bad resolutions. As a result, a mixture of methanol, acetonitrile and KH_2_PO_4_ buffer at pH 5 was considered to achieve adequate separation.

Traditionally, designing chromatographic systems involved applying a large number of steps to achieve the best required results, a process known as Univariate optimization which involved examining one variable at a time and keeping the other ones constant, a laborious and time-consuming process. To tackle this problem, full factorial design approach was used.

Consequently, a factorial design with two levels and three independent factors was used for method testing and optimization. The factors studied were the percent of methanol and acetonitrile and the concentration of the buffer used, chosen as the most important factors affecting the HPLC method. Methanol ratios of 20 and 35 were tested, acetonitrile ratios of 15 and 20 were tested and buffer concentrations of 0.02 and 0.05 M were tested. As a result, the higher and lower levels were assigned the values of −1 and + 1, respectively, with the three independent parameters being methanol ratio (X1), acetonitrile ratio (X2) and buffer concentrations (X3)^51^. Testing the significance of the independent factors was possible using an approximated Fisher Statistical Test for Variance Analysis (ANOVA) model which was utilized to test the effect of the independent factors on the responses in addition to their interactions^52^(**Table ****S1**). The equation representing the three-factor design is found to be:

1 $$Y = ~\beta ^\circ + ~\beta 1X1 + ~\beta 2X2 + ~\beta 3X3 + \beta 12\left( {X1X2} \right) + ~\beta 13\left( {X1X3} \right) + ~\beta 23\left( {X2X3} \right) + ~\beta 123\left( {X1X2X3} \right)$$

Where Y represents the experimental response, βº the intercept and β1–β123 are the coefficients for the factors X1 (methanol), X2 (acetonitrile) and X3 (buffer concentration).

The regression equation for resolution was found to be:

Rs = 17.6–0.346 MeOH − 0.154 ACN − 4.1 Buffer conc.

and for the run time to be:

Run time = 20.2–0.302 MeOH − 0.242 ACN + 7.5 Buffer conc.

The optimum conditions giving the best responses are judged based on the composite desirability of a response (D) determined by the Minitab response optimizer. The values lie between zero and 1, with zero meaning that the responses are outside the acceptable ranges and 1 indicating the ideality of the conditions. So, the closer the values are to 1 the better.

Pareto charts and half normal plots are obtained which are useful in determining which factor has the most significant effect on the measured parameters (**Fig. ****S1**** and S2**).

Figure S2 shows that methanol had a significant effect on both resolution and run time, with as a statistical significant effect for 95% confidence level.

**Fig. 3:**
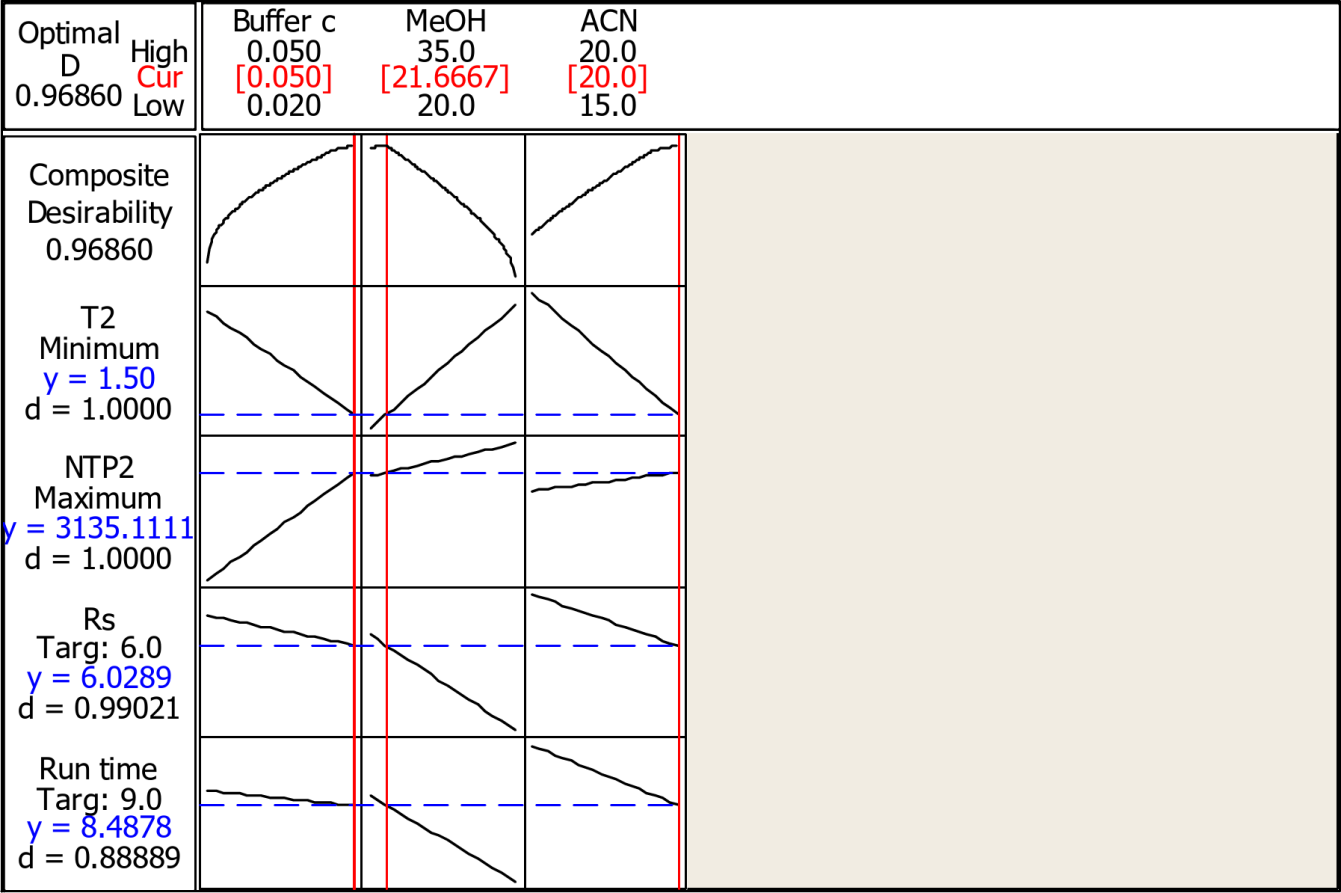
2^3^ full factorial design optimization plot for the proposed HPLC method.

**Fig. 4:**
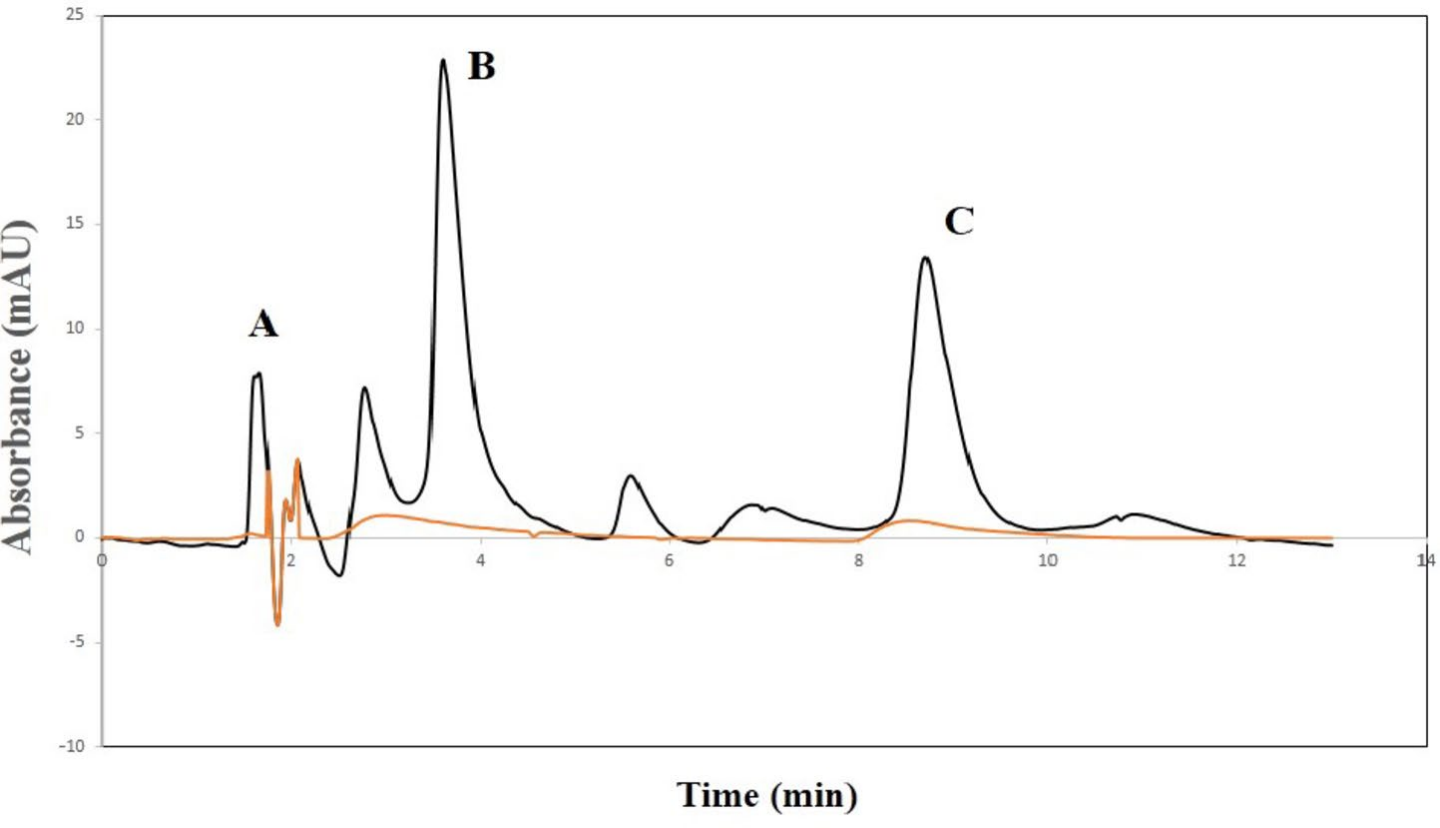
Representative chromatogram of 15.0 µg/mL meloxicam (**B**) and 20.0 µg/mL esomeprazole (**C**) under the optimum conditions, with (**A**) being the solvent front (Brown chromatogram represents the mobile phase only).

Moreover, other significant results were obtained from the main effect and interaction plots (**Figures S3 and S4**) which indicated significant interaction between methanol and buffer concentration as well as showing that reaching the optimum results can be achieved using methanol: acetonitrile: 0.05 M phosphate buffer in the ratio of 20: 20: 60 to reach optimum separation of both drugs that confirmed after 5 trials as shown in **Table S2 and S3.** Experimental design was utilized to obtain the optimum results, with the optimization plot showing the variables and the interactions affecting the composite desirability and response (Figs. 3 and 4).

The results of optimization are shown in Tables 1 and 2; Fig. 3, which were compared with pharmacopoeial values^4^.

**Table 1:** System suitability parameters of the proposed HPLC method.*Reference values are: K’: 0–10, α > 1.0, R_s_ > 1.5 and T: 0.9–1.2 ^4^.

Parameters*	MEL	EPL
No. of theoretical plates (N)	493	1392
Capacity factor, k’	1.17	4.23
Selectivity factor, α	3.62	
Resolution (R_s_)	6.80	
Retention time (t_R_), min	3.62	8.72
Tailing factor	2.61	1.34

**Table 2:** Performance data of the proposed HPLC method.

Parameters	MEL	EPL
Linearity (µg/mL)	5.0–100.0	10.0–100.0
LOD (µg/mL)	0.86	1.82
LOQ (µg/mL)	2.62	5.52
r	0.9999	0.9999
Slope	25.57	30.91
Intercept	−18.24	−212.39
S_y/x_	9.90	19.64
S_a_	6.69	17.06
S_b_	0.12	0.28
% Error	0.62	0.65
% RSD	1.51	1.45
Mean Found (%) ± SD	100.41 ± 1.51	100.57 ± 1.46

### Analytical performance and validation of the proposed method

#### Range and linearity

The linearity of the proposed HPLC method was found to be 5.0–100.0 µg/mL and 10.0–100.0 µg/mL for MEL & EPL, respectively, with regression coefficients equal to 0.99995 & 0.9999 for meloxicam & esomeprazole, respectively.

#### Limits of detection and quantification

The LOD and LOQ values were calculated according to the following Eqs. ^53,54^:

$$\:LOD=\frac{3.3\sigma\:}{S}\:\:\:\&\:\:\:\text{LOQ}=\frac{10{\upsigma\:}}{\text{S}}$$

Where σ is the standard deviation of the response and S is the slope of the calibration curve calculated from the regression line.

#### Precision and accuracy

Accuracy was estimated as per ICH recommendations^53^. For MEL, it was estimated *via* spectrophotometry utilizing its zero order measurement in 0.1 M NaOH using λmax = 269 nm^55^. For EPL, it was determined spectrophotometrically in methanol using its first order measurement at 299.5 nm (∆λ = 8, scaling factor = 1)^32^. The methods were also applied to the dosage forms revealing no statistical difference, indicated by the t-test & F-values, shown in Table 3. Inter and intraday precisions were done according to ICH guidelines^53^ requiring the determination of three concentrations within the studied ranges on three successive times or three successive days as shown in Table 4 and Table 5.

**Table 3:** Statistical comparison between the proposed HPLC method and the reference methods for MEL and EPL.

Parameters	MEL		EPL	
	Proposed method**	Reference method ^55^	Proposed method**	Reference method ^32^
Prepared tablets				
Mean % Recoveries ± SD	100.42 ± 0.99	99.80 ± 1.72	100.72 ± 1.31	99.08 ± 1.40
% Error	0.50	0.99	0.65	0.82
% RSD	0.99	1.72	1.30	1.42
t-test	0.61 (2.57)*		1.59 (2.57)*	
F-value	2.99 (9.55)*		1.15 (9.55)*	
Pure Drugs				
Linearity range (µg/mL)	5.0–100.0	5.0–30.0	10.0–100.0	2.0–10.0
Mean % Recoveries ± SD	100.41 ± 1.51	100.01 ± 0.73	100.57 ± 1.46	99.91 ± 0.58
% Error	0.62	0.33	0.65	0.26
% RSD	1.51	0.73	1.45	0.58
t-test	0.54 (2.26)*		0.94 (2.31)*	
F-value	4.32 (6.26)*		6.32 (6.39)*	

* The values in parentheses indicate the tabulated t- and F-values at P = 0.05 ^56^.

** The mean % recoveries are calculated for 3 concentrations.

**Table 4:** Intraday precision of the proposed HPLC method.

Parameters	MEL		EPL	
	Amount taken (µg/mL)	% Recoveries *	Amount taken (µg/mL)	% Recoveries *
	15.0	100.27	20.0	101.25
		98.02		100.14
		97.87		99.10
	Mean % ± SD	98.72 ± 1.34	Mean % ± SD	100.16 ± 1.08
	% Error	0.79	% Error	0.62
	% RSD	1.36	Mean % ± SD	1.08
	20.0	99.24	25.0	100.40
		99.43		98.65
		98.59		98.96
	Mean % ± SD	99.09 ± 0.44	Mean % ± SD	99.34 ± 0.93
	% Error	0.26	% Error	0.54
	% RSD	0.45	% RSD	0.94
	25.0	97.14	30.0	97.94
		99.36		98.21
		98.98		99.61
	Mean % ± SD	98.49 ± 1.19	Mean % ± SD	98.59 ± 0.90
	% Error	0.70	% Error	0.53
	%RSD	1.21	% RSD	0.91

* Average of three determinations for each concentration.

**Table 5:** Intraday precision of the proposed HPLC method.

Parameters	MEL		EPL	
	Amount taken (μg/mL)	% Recoveries *	Amount taken (μg/mL)	% Recoveries *
	15.0	98.01	20.0	98.58
		101.72		101.84
		100.28		100.70
	Mean % ± SD	100.00 ± 1.87	Mean % ± SD	100.37 ± 1.66
	% Error	1.08	% Error	0.95
	% RSD	1.87	% RSD	1.65
	20.0	98.76	25.0	100.66
		98.98		101.28
		100.71		98.87
	Mean % ± SD	99.48 ± 1.07	Mean % ± SD	100.27 ± 1.25
	% Error	0.62	% Error	0.72
	% RSD	1.07	% RSD	1.25
	25.0	99.23	30.0	98.88
		99.40		100.97
		100.53		100.72
	Mean % ± SD	99.72 ± 0.71	Mean % ± SD	100.19 ± 1.14
	% Error	0.41	% Error	0.66
	%RSD	0.71	% RSD	1.14

* Average of three determinations for each concentration.

#### Robustness

The robustness of the method was established by deliberate slight variations in the chromatographic conditions such as pH (5 ± 0.2), acetonitrile (20 ± 0.2), methanol (20 ± 0.2), buffer volume (60 ± 0.2) & flow rate (1 mL/min ± 0.1). The changes had minor effects on the resolution, indicating the robustness as shown in Table 6.

**Table 6:** Robustness of the proposed HPLC method *.

Parameter	Values	Resolution	NTP (MEL)	NTP (EPL)
pH (± 0.2)	4.8	6.75	488	1387
	5	6.8	493	1392
	5.2	6.79	490	1395
Acetonitrile %	19.8	6.69	491	1396
	20	6.8	493	1392
	20.2	6.76	490	1389
Methanol %	19.8	6.81	493	1390
	20	6.8	493	1392
	20.2	6.82	487	1386
Buffer %	59.8	6.77	491	1391
	60	6.8	493	1392
	60.2	6.79	495	1388
Flow rate (mL/min)	0.9	6.79	491	1391
	1	6.8	493	1392
	1.1	6.8	491	1390

* The chosen concentrations were 15&nbsp;µg/mL and 20&nbsp;µg/mL for MEL and EPL, respectively. Each experiment was repeated 3 times for each parameter tested.

### Application to laboratory-prepared tablets

The proposed method was applied to the laboratory prepared tablets. Statistical comparison revealed no significant differences between the proposed method and the reference methods, indicated by the values of t-test and F-test. The results are summarized in Table 2 and Table 7.

**Table 7:** Application of the proposed method to the laboratory prepared tablets of meloxicam & Esomeprazole.

Parameters	MEL		EPL	
	**Amount taken (µg/mL)**	% Recoveries *	**Amount taken (µg/mL)**	% Recoveries *
	15.0	99.04	20.0	98.81
	20.0	101.31	26.7	101.58
	25.0	100.95	33.3	101.58
	30.0	100.38	40.0	100.90
Mean % ± SD	100.42 ± 0.99		100.72 ± 1.31	
% Error	0.50		0.65	
% RSD	0.99		1.30	

* Average of three determinations for each concentration.

### Evaluation of the method greenness

One of the most important goals in analytical chemistry is the design of an analytical technique that possesses the characteristics of being simple, inexpensive and uses simple, easily-operated and available instruments. However, an extremely important factor should be taken into consideration, which is the effect of this technique on the environment to comply with the principles of green chemistry which necessitates reducing the use and emission of toxic compounds and reagents. Thus thorough evaluation of the method greenness-wise is a must to ensure the conformity to the requirements of green chemistry^57^. Four methods were used to evaluate the method greenness. The first one is the analytical eco-scale method which assigns penalty points to each step not abiding to the principles of the ideal green analysis and calculates a greenness score using the following Eq. 5^7^: Analytical Eco-Scale = 100 − total penalty points. The results are summarized in Table 8.

**Table 8:** Calculation of penalty points for the proposed method using the analytical Eco-Scale method.

The penalty points (PPs) calculation according to analytical Eco-Scale		Penalty points
Reagents		
Methanol	Amount 10–100 mL	12
Acetonitrile	Amount 10–100 mL	8
Phosphate buffer	Amount 10–100 mL	0
Instrument		
Energy	Technique: LC ⩽1.5 kWh per sample	1
Occupational hazard	Analytical process hermetization	0
Waste	˃ 10 mL (g)	5
Waste treatment	No treatment	3
Total penalty points		29
Analytical Eco-Scale total score *		71 *

* Score ranking scale: >75: excellent green analysis, >50: acceptable green analysis, <50: inadequate green analysis^45^.

**Fig. 5:**
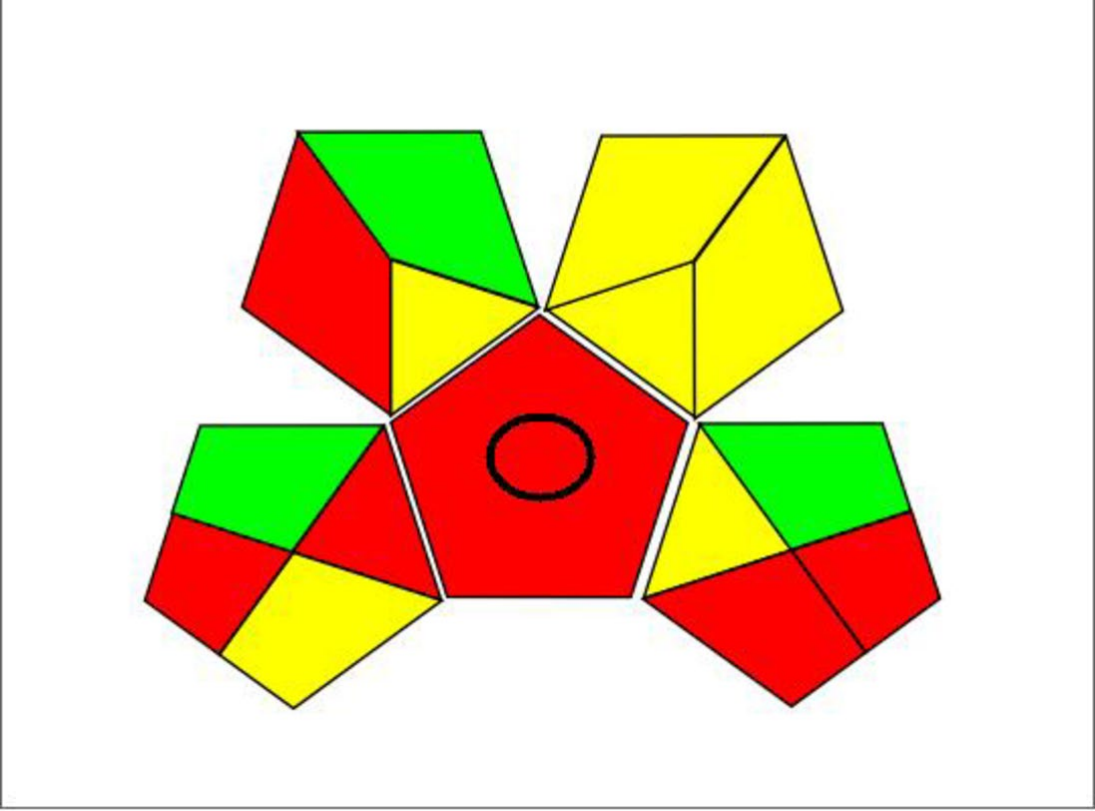
GAPI pictograms used to assess the greenness of the proposed method.

The second method used is the Green Analytical Procedure Index (GAPI) technique which assesses the environmental impacts of the analytical method by the means of five pictograms, offering several additional advantages not covered by the previous method, such as sample processing and preparation in addition to the chemical nature of reagents used^57^. The resultant GAPI chart is shown in Fig. 5.

**Fig. 6:**
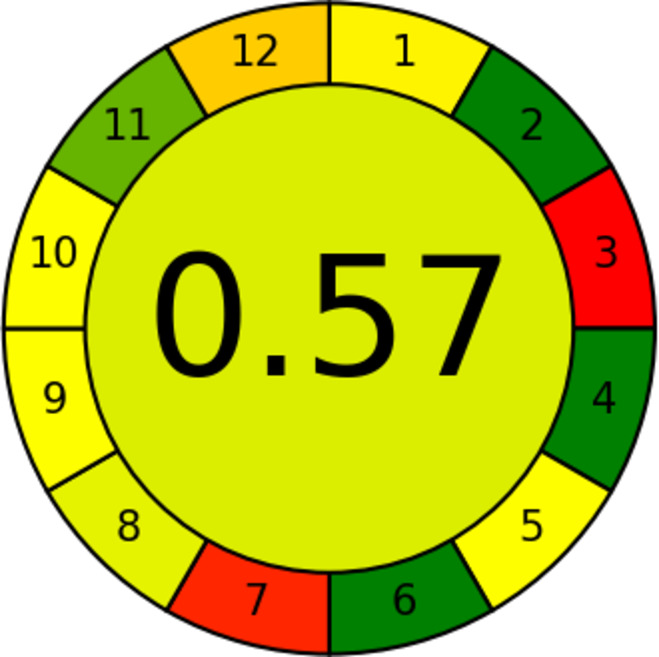
AGREE method used for estimating the greenness of the proposed method.

The third method used is the Analytical Greenness Calculator (AGREE) technique. It gives the extra merit of abiding by all the principles of green chemistry in addition to the production of the final result in both color and numeric form^57^. The results obtained are shown in Fig. 6.

The fourth method used is a method used specifically for HPLC techniques, which is HPLC Environment Assessment chart (HPLC-EAT) method. The method takes into account all solvents used in the HPLC technique with respect to safety, health and environmental impact and calculates a score judging the greenness of the whole analytical technique based on the following Eq. 5^58^:

$$\text{HPLC-EAT} = \text{S}_1\text{m}_1 + \text{H}_1\text{m}_1 + \text{E}_1\text{m}_1 + \text{S}_2\text{m}_2 + \text{H}_2\text{m}_2 + \text{E}_2\text{m}_2 +....+ \text{S}_\text{n}\text{m}_\text{n} + \text{H}_\text{n}\text{m}_\text{n} + \text{E}_\text{n}\text{m}_\text{n}$$

**Fig. 7:**
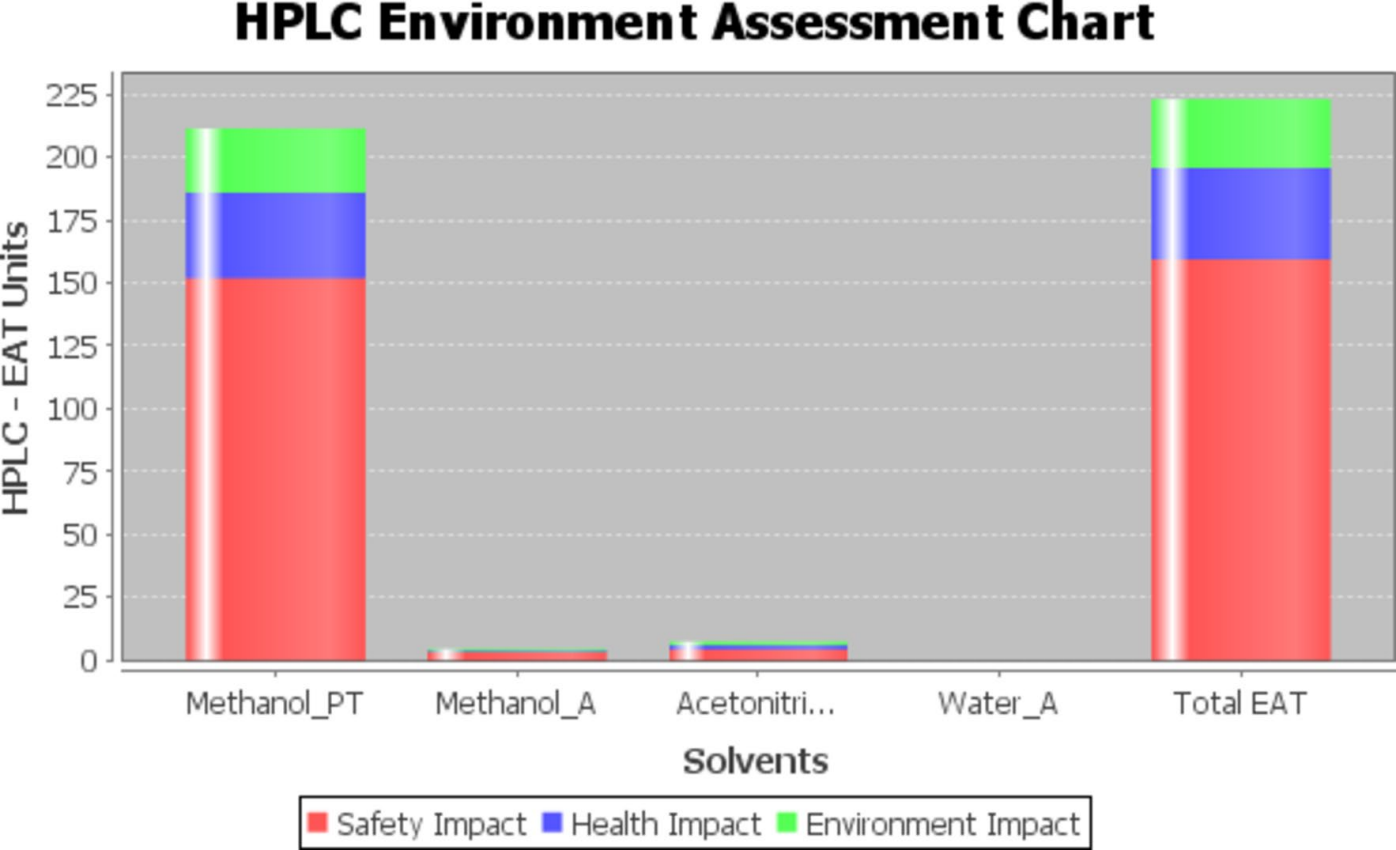
HPLC-EAT chart for the proposed method.

Where S, H, and E are safety, health and environmental factors, respectively for n number of solvents, and m is the mass of the solvent(s). The method is considered green at low scores. While the method doesn’t provide a reference range regarding the score greenness, it is nevertheless important for comparing different HPLC methods and thus is included as a reference for future work. The software can be downloaded free of charge and is provided in the supplementary section of the reference^58^. The Total EAT score calculated is 220 and the resultant chart is in Fig. 7.

## Conclusion

An HPLC method combined with full factorial design was utilized for the first time for the simultaneous estimation of meloxicam and esomeprazole in their tablet dosage forms. A multivariate investigation of the process variables; % of methanol, % of acetonitrile and concentration of buffer was conducted to reach the optimum system for separation. Four methods were used to assess the method greenness and acceptable greenness was concluded from the results. The whole chromatographic process duration time was less than 10 min. The proposed method is, thus, fast, simple & reproducible with acceptable range, accuracy & precision in addition to being of acceptable greenness, making it useful for the routine quality control of the dosage form.

## Electronic supplementary material

Below is the link to the electronic supplementary material.

Supplementary Material 1

## Data Availability

Data availability statement: All data generated and/or analyzed during this study are included in this published article.
