# Supplementary material for: Deduction of full factorial design of HPLC technique for the simultaneous analysis of meloxicam and esomeprazole in their laboratory prepared tablets
Source: Sci Rep. 2025 Apr 15;15:12922. doi: 10.1038/s41598-025-95706-3 (PMC12000416; doi:10.1038/s41598-025-95706-3)
Supplement: Supplementary file 1 — Supplementary Material 1 [file 41598_2025_95706_MOESM1_ESM.docx]

**Supplementary data**

**Deduction of full factorial design of HPLC technique for the simultaneous analysis of meloxicam and esomeprazole in their laboratory prepared tablets**

Hesham Sameh Ramadan ^a,b
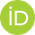
^, Fathalla Belal **^c^**, Aya Roshdy **^b^,** Mohamed M. Salim **^b,c,^**^*^

**^a^** Department of Pharmaceutical Analytical Chemistry, Faculty of Pharmacy, Suez Canal University, Ismailia, Egypt

**^b^** Department of Pharmaceutical Chemistry, Faculty of Pharmacy, Horus University- Egypt, New Damietta, Egypt

^c^ Department of Pharmaceutical Analytical Chemistry, Faculty of Pharmacy, Mansoura University, Mansoura 35516, Egypt

^*^ Correspondence: Department of Pharmaceutical Analytical Chemistry, Faculty of Pharmacy, Mansoura University, Mansoura 35516, Egypt.

Department of Pharmaceutical Chemistry, Faculty of Pharmacy, Horus University - Egypt, New Damietta, Egypt.

**E-mail address: mmasalim@mans.edu.eg**

**Figure Caption:**

**Fig. S1: Half normal plots ………………………………………..…….……….3**

**Fig. S2: Interaction plots ………………………………………………..……….3**

**Fig. S3: Pareto charts…………………………………………………………….4**

**Fig. S4: Main effects plots……………………………………………..………….4**

**Fig. S5: Contour and surface plots…………………………..…………..……….5**

**Fig. S6: Box-Cox plots…………….…………………………..………….……….5**

**Fig. S1: Half normal plots**

**
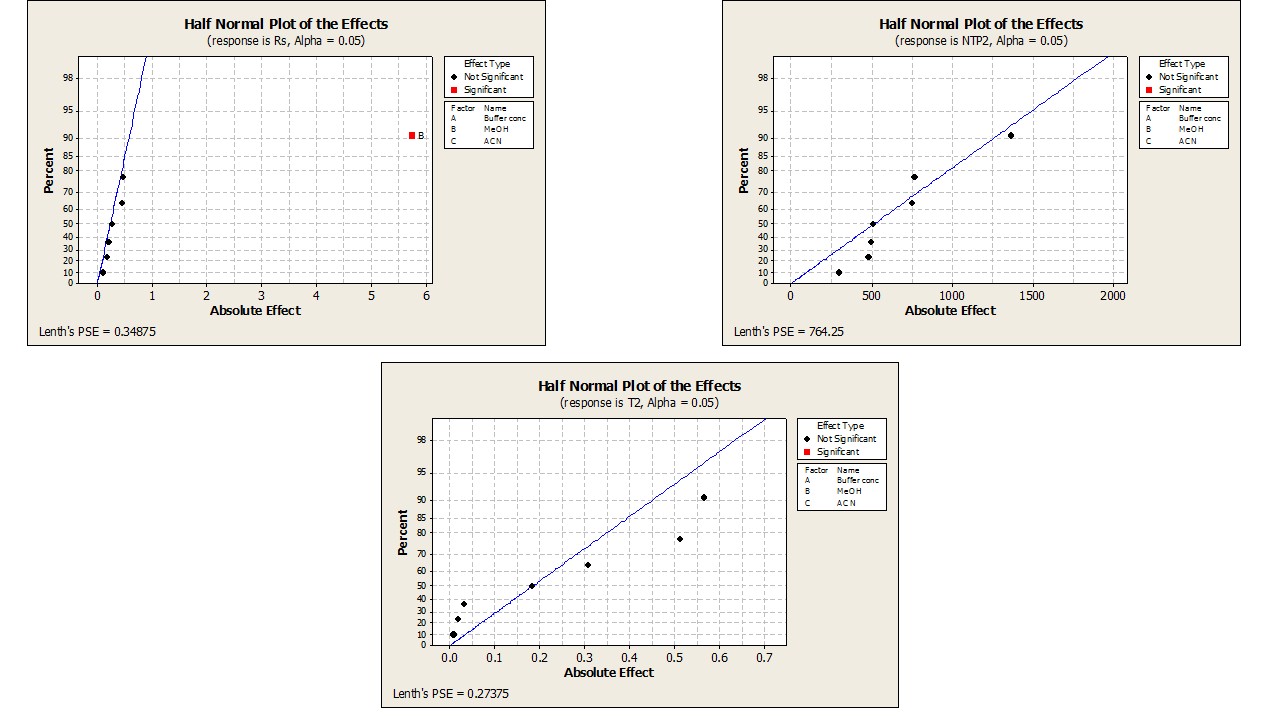
**

**Fig. S2: Interaction plots**

**
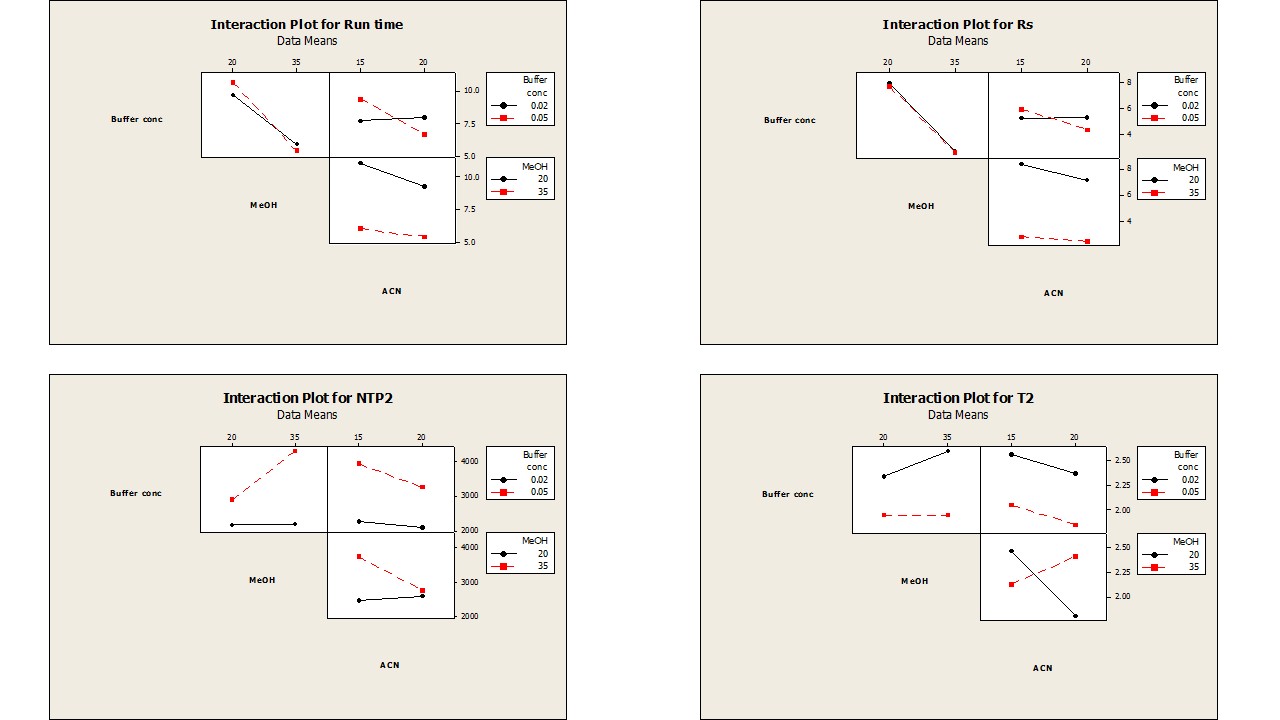
**

**Fig. S3: Pareto Charts**

**
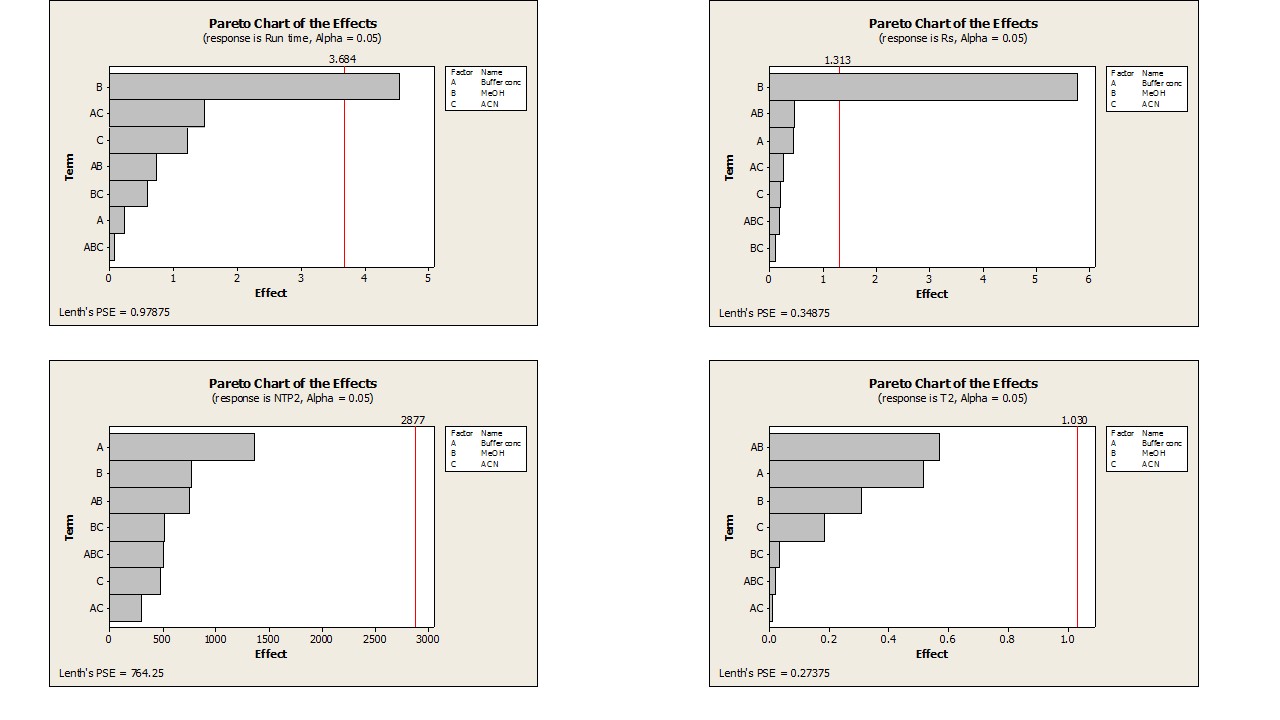
**

**Fig. S4: Main effects plots**

**
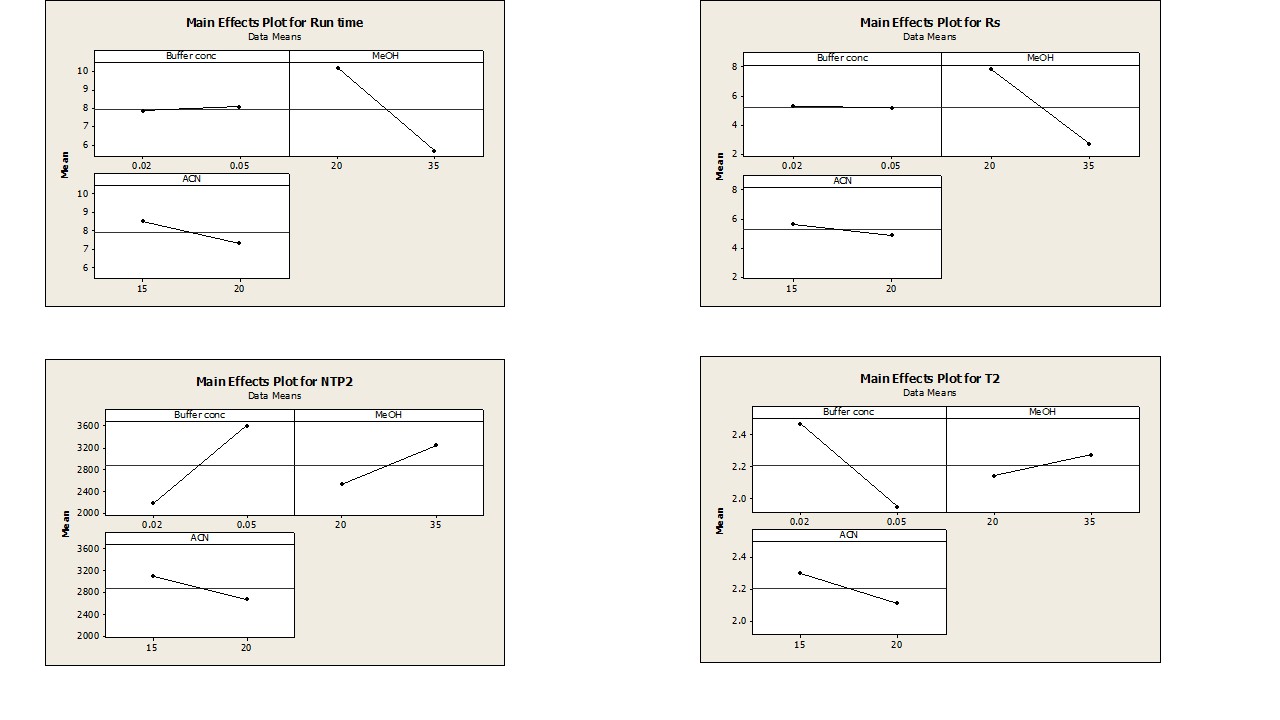
**

**Fig. S5: Contour and surface plots**

**
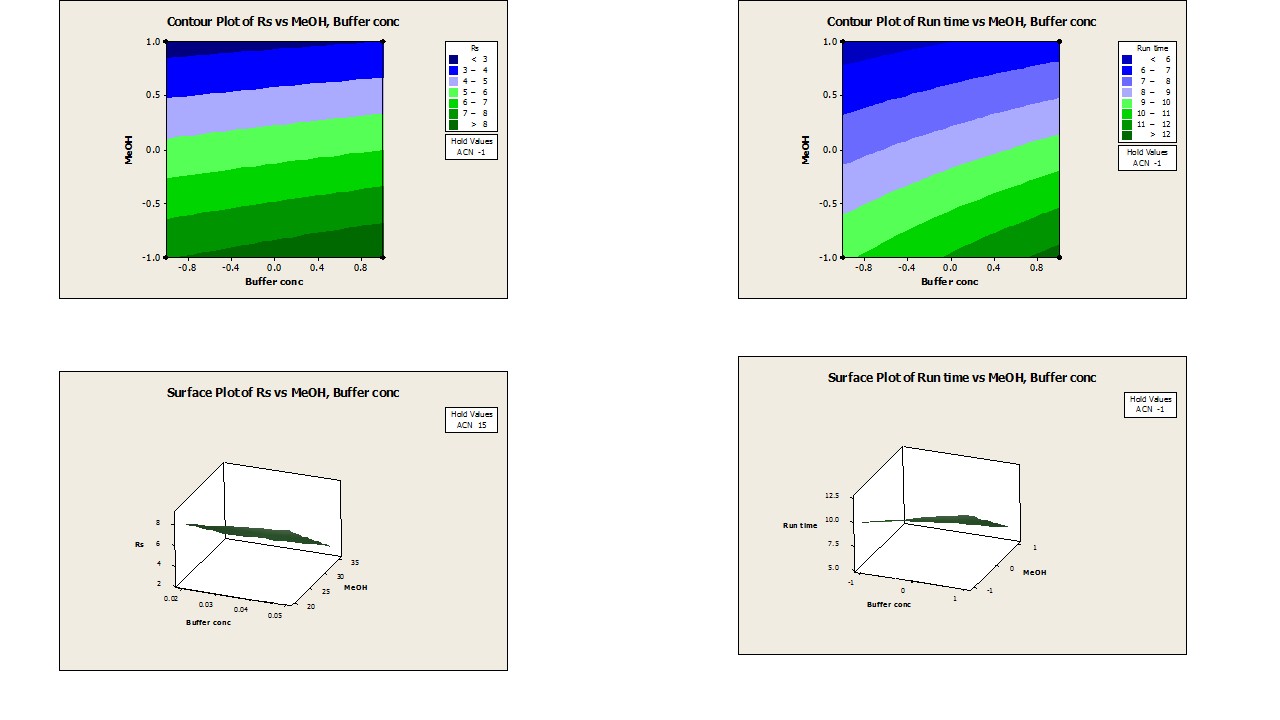
**

**Fig. S6: Box-cox plots**

**List of Tables:**

**Table S1: ANOVA results……………………………………………………..7**

**Table S2: Measured responses………………………………………………..8**

**Table S3: Trials under optimum conditions………………………………….8**

**Table S4: Response Optimization……………………………………………8-9**

**Table S1: ANOVA results**

**Two-way ANOVA: Rs versus Buffer conc, MeOH**

| Source | DF | SS | MS | F | P |
| --- | --- | --- | --- | --- | --- |
| Buffer conc | 1 | 0.0300 | 0.0300 | 0.04 | 0.858 |
| MeOH | 1 | 53.9241 | 53.9241 | 65.69 | 0.001 |
| Interaction | 1 | 0.0190 | 0.0190 | 0.02 | 0.886 |
| Error | 4 | 3.2836 | 0.8209 |  |  |
| Total | 7 | 57.2567 |  |  |  |

S = 0.9060, R-Sq = 94.27%, R-Sq (adj) = 89.96%

**Two-way ANOVA: Run time versus Buffer conc, MeOH**

| Source | DF | SS | MS | F | P |
| --- | --- | --- | --- | --- | --- |
| Buffer conc | 1 | 0.1012 | 0.1012 | 0.05 | 0.834 |
| MeOH | 1 | 41.0418 | 41.0418 | 20.32 | 0.011 |
| Interaction | 1 | 1.0513 | 1.0513 | 0.52 | 0.511 |
| Error | 4 | 8.0795 | 2.0199 |  |  |
| Total | 7 | 50.2738 |  |  |  |

S = 1.421, R-Sq = 83.93%, R-Sq (adj) = 71.88%

**Analysis of Variance for Run time (coded units)**

| Source | DF | Seq SS | Adj SS | Adj MS |
| --- | --- | --- | --- | --- |
| Main Effects | 3 | 44.0712 | 44.0712 | 14.6904 |
| Buffer conc | 1 | 0.1012 | 0.1012 | 0.1012 |
| MeOH | 1 | 41.0418 | 41.0418 | 41.0418 |
| ACN | 1 | 2.9282 | 2.9282 | 2.9282 |
| 2-Way Interactions | 3 | 6.1941 | 6.1941 | 2.0647 |
| Buffer conc*MeOH | 1 | 1.0512 | 1.0512 | 1.0512 |
| Buffer conc*ACN | 1 | 4.4700 | 4.4700 | 4.4700 |
| MeOH*ACN | 1 | 0.6728 | 0.6728 | 0.6728 |
| 3-Way Interactions | 1 | 0.0084 | 0.0084 | 0.0084 |
| Buffer conc*MeOH*ACN | 1 | 0.0084 | 0.0084 | 0.0084 |
| Residual Error | 0 |  |  |  |
| Total | 7 | 50.2738 |  |  |

**Analysis of Variance for Rs (coded units)**

| Source | DF | Seq SS | Adj SS | Adj MS |
| --- | --- | --- | --- | --- |
| Main Effects | 3 | 66.7712 | 66.7712 | 66.7712 |
| Buffer conc | 1 | 0.3916 | 0.3916 | 0.3916 |
| MeOH | 1 | 66.2976 | 66.2976 | 66.2976 |
| ACN | 1 | 0.0820 | 0.0820 | 0.0820 |
| 2-Way Interactions | 3 | 0.5959 | 0.5959 | 0.1986 |
| Buffer conc*MeOH | 1 | 0.4371 | 0.4371 | 0.4371 |
| Buffer conc*ACN | 1 | 0.1378 | 0.1378 | 0.1378 |
| MeOH*ACN | 1 | 0.0210 | 0.0210 | 0.0210 |
| 3-Way Interactions | 1 | 0.0595 | 0.0595 | 0.0595 |
| Buffer conc*MeOH*ACN | 1 | 0.0595 | 0.0595 | 0.0595 |
| Residual Error | 0 |  |  |  |
| Total | 7 | 67.4267 |  |  |

**Table S2: Measured responses**

| **Buffer** | **MeOH** | **ACN** | **T2** | **NTP2** | **Rs** | **Run time** |
| --- | --- | --- | --- | --- | --- | --- |
| 0.02 | 35 | 15 | 2.67 | 2290 | 2.59 | 5.52 |
| 0.02 | 20 | 15 | 2.46 | 2262 | 7.95 | 9.84 |
| 0.05 | 20 | 15 | 2.49 | 2672 | 8.95 | 12.35 |
| 0.02 | 20 | 20 | 2.22 | 2088 | 7.94 | 9.61 |
| 0.02 | 35 | 20 | 2.53 | 2095 | 2.72 | 6.32 |
| 0.05 | 35 | 15 | 1.60 | 5198 | 3.00 | 6.45 |
| 0.05 | 35 | 20 | 2.30 | 3416 |  | 4.39 |
| 0.05 | 20 | 20 | 1.40 | 3100 | 6.50 | 9.00 |

**Table S3: Trials under optimum conditions**

|  | **Mean** | **SD** | **% Error** |
| --- | --- | --- | --- |
| **T2** | 99.10 | 0.46 | 0.27 |
| **NTP** | 99.47 | 1.50 | 0.87 |
| **Rs** | 99.90 | 0.30 | 0.17 |
| **Run time** | 99.53 | 0.68 | 0.39 |

**Table S4: Response Optimization**

| Parameters | Goal | Lower | Target | Upper | Weight | Import |
| --- | --- | --- | --- | --- | --- | --- |
| T2 | Minimum | 1.50 | 1.5 | 2.67 | 1 | 1 |
| NTP2 | Maximum | 2088.00 | 3000.0 | 3000.0 | 1 | 1 |
| Rs | Target | 2.26 | 6.0 | 8.95 | 1 | 1 |
| Run time | Target | 4.39 | 9.0 | 12.35 | 1 | 1 |

**Starting Point**

Buffer conc = 0.02

MeOH = 20

ACN = 15

**Local Solution**

Buffer conc = 0.05

MeOH = 21.6667

ACN = 20

**Predicted Responses**

T2 = 1.50, desirability = 1.000000

NTP2 = 3135.11, desirability = 1.000000

Rs = 6.03, desirability = 0.990207

Run time = 8.49, desirability = 0.888889

**Composite Desirability** = 0.968598
